# Circular RNA GLIS2 promotes colorectal cancer cell motility via activation of the NF-κB pathway

**DOI:** 10.1038/s41419-020-02989-7

**Published:** 2020-09-23

**Authors:** Junxiong Chen, Xiangling Yang, Ruixian Liu, Chuangyu Wen, Huihui Wang, Lanlan Huang, Weiqian Li, Zefeng Zhu, Yonglin Zhu, Huanliang Liu

**Affiliations:** 1 Department of Clinical Laboratory, The Sixth Affiliated Hospital, Sun Yat-sen University, Guangzhou, Guangdong China; 2 Guangdong Provincial Key Laboratory of Colorectal and Pelvic Floor Diseases, Guangdong Institute of Gastroenterology, The Sixth Affiliated Hospital, Sun Yat-sen University, Guangzhou, Guangdong China

**Keywords:** Colorectal cancer, Oncogenes

## Abstract

Circular RNAs (circRNAs) are a newly discovered type of biological molecule that belongs to the noncoding RNA family. Abundant evidence has shown that circRNAs are involved in the progression of various cancers. However, the particular functions of circRNAs in colorectal cancer (CRC) remain elusive. In this study, we investigated the differentially expressed circRNAs in three pairs of cancer tissue and adjacent normal tissue of CRC. We revealed that circGLIS2 expression was higher in CRC tissue and cell lines. Gain-and-loss function assays showed that circGLIS2 was involved in the regulation of cell migration. Moreover, overexpressing circGLIS2 in CRC cells activated the NF-κB pathway and induced pro-inflammatory chemokine production, which evoked tumor-associated inflammation through recruiting leukocytes. In turn, when the cancer cells were exposed to the supernatant of circGLIS2 overexpressed cancer cells, they were endowed with the ability of migration and chemokines production. Furthermore, the rescue assay confirmed that circGLIS2 activated NF-κB signaling and promoted cell migration by sponging miR-671. Overall, our study reveals that circGLIS2, acting as a potential oncogene, maintains the abnormal activation state of the NF-κB signaling pathway via the miR-671 sponge mechanism in CRC cells. This study provides a scientific basis for targeting circGLIS2 in colorectal cancer interventions.

## Introduction

Colorectal cancer (CRC) is the third most common malignancy and the fourth most common cause of death in cancers around the world^1^. With the development of early diagnosis and treatment, the survival rate of CRC has increased over the past few years in the United States^2^. Nevertheless, tumor metastasis is still a major factor hindering the therapeutic efficacy of CRC treatments. Therefore, studying the underlying molecular mechanism of CRC metastasis is highly important to improve CRC treatment.

Circular RNAs (circRNAs), characterized by their back-spliced ring structure, are widely expressed in eukaryotes^3,4^. Growing evidence has indicated that circRNAs participate in the metastasis of many kinds of cancers^5–7^. Structurally, circRNAs are more stable than other kinds of RNAs, because of their tolerance of exonucleases. Functionally, circRNAs act as miRNAs sponges to regulate the expression of target genes via the miRNAs response element^8,9^. This notion has been supported by a series of previous studies: circPVT1 functions as an oncogene to promote metastasis via miR-145 sponging in CRC^10^; circPDE8A promotes the invasive growth of pancreatic ductal adenocarcinoma cells by antagonizing miR-338 to enhance the MET pathway^11^; and fibroblast growth factor receptor 1 (FGFR1) derived circular RNA circFGFR1 promotes non-small cell lung cancer progression and resistance to immune checkpoint inhibitor therapy via miR-381-3p/CXCR4 axis^12^.

In this study, to explore the differentially expressed circRNAs in CRC development, we analyzed levels of circRNAs in paired CRC tissue and adjacent normal tissue by microarray. We identified that circGLIS2 is upregulated in CRC tissue and cell lines. We provide evidence that circGLIS2 promotes CRC cells migration by activating the NF-κB signaling pathway. We further showed that the supernatant collected from circGLIS2 overexpressed cells can activate the NF-κB signal pathway and promote cell motility. Furthermore, we observed that more leukocytes were recruited to the supernatant of circGLIS2 overexpressed cells compare to the vector control cells. Mechanically, circGLIS2 sponge miR-671 to modulate NF-κB signaling pathway and consequently enhance the motility of CRC cells. These results indicated that circGLIS2/miR-671/NF-κB pathway axis regulated metastasis function via an autocrine-paracrine manner in CRC, which may be a promising target for CRC intervention.

## Results

### CircGLIS2 is upregulated in CRC

**Fig. 1:**
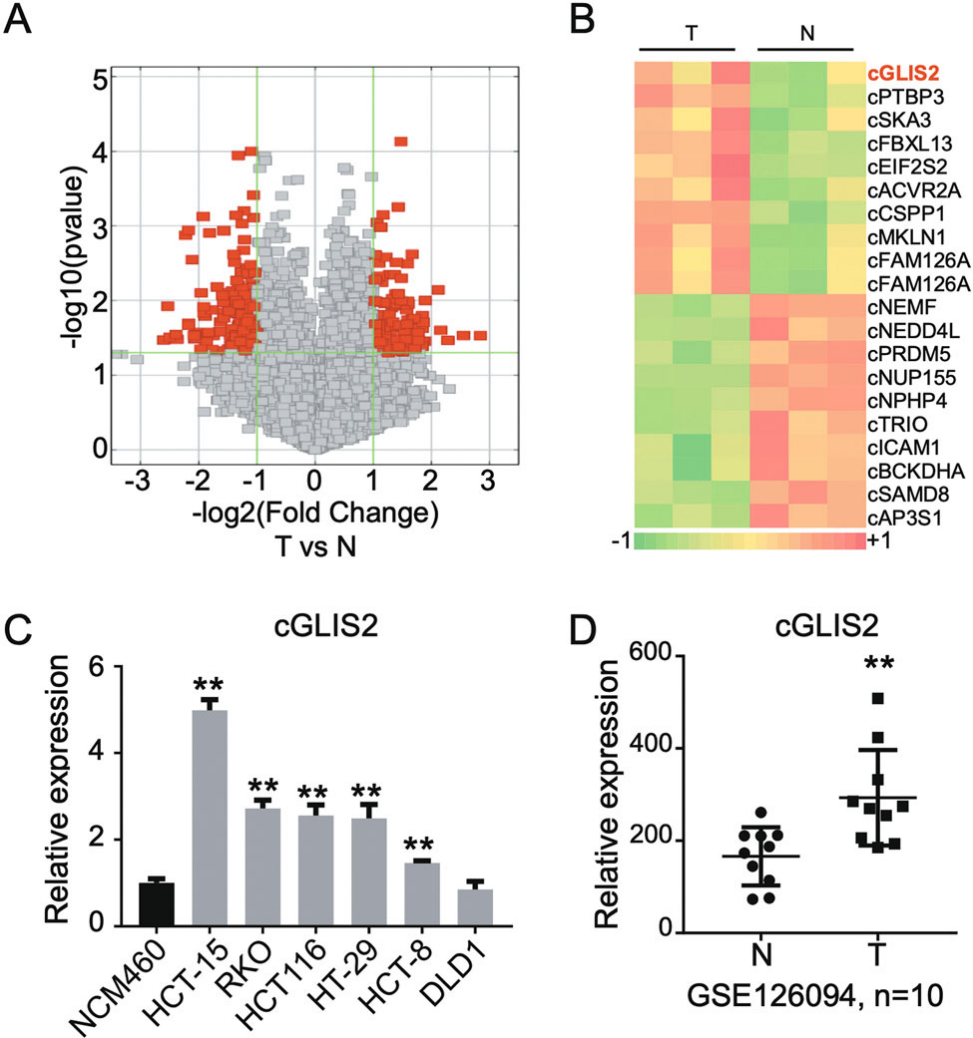
CircGLIS2 is overexpressed in colorectal cancer. **a** Volcano plot describing the profile of circRNAs expression in CRC tissue and adjacent normal tissue. Significantly changed circRNAs are marked with red dots (*P* < 0.05, fold change > 2.0). **b** The heatmap for top 20 differentially expressed circRNAs in three cases of clinical samples, with rows referring to circRNAs and columns referring to the samples. **c** qRT-PCR comparing the expression of circGLIS2 in various colorectal cancer cell lines and NCM460 representing normal colon cell lines. **d** Scatter plots illustrating the expression fold change for circGLIS2 in CRC tissue compared with adjacent normal tissue from the GSE126094 database (*n* = 10). Data are represented as mean ± SD. **P < 0.01.

CircRNAs plays an important role in many diseases, including cancer. To identify the circRNAs that is closely related to CRC, three pairs of human CRC clinical samples and adjacent normal tissue (Table S1) were analyzed using a circRNA microarray assay. As shown in Fig. 1a, the volcano plot described 305 circRNAs that were differentially expressed in CRC (135 upregulated and 170 downregulated). And the heatmap showed the top 20 differentially expressed circRNAs in CRC (Fig. 1b). CircGLIS2 (has_circRNA_101692, probe ID: ASCRP002039) was upregulated in the CRC samples and has not been studied in cancer yet. The level of circGLIS2 was quantified in various CRC cell lines. As shown in Fig. 1c, the expression of circGLIS2 was increased in CRC cell lines compared to the NCM460 colon normal cell line. To verify the research value of circGLIS2 in CRC, a CRC-related circRNAs dataset (GSE126094) was attached. As shown in Fig. 1d, circGLIS2 was significantly upregulated in CRC samples compared with adjacent normal control.

### CircGLIS2 is generated from the GLIS2 gene by back-splicing

**Fig. 2:**
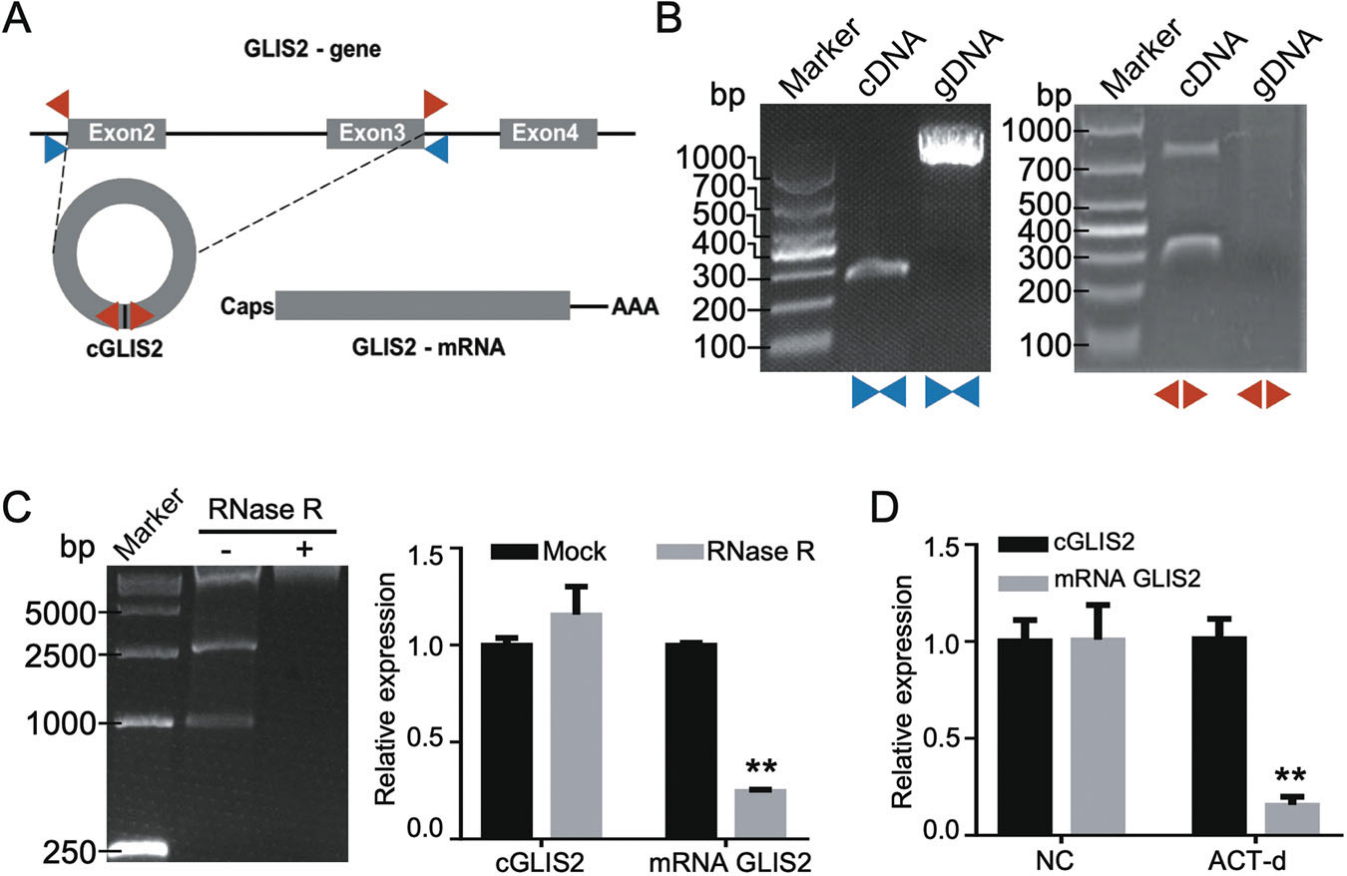
CircGLIS2 is generated from exon 2 and 3 of the GLIS2 gene by back-splicing. **a** Schematic shows circGLIS2 is generated from exon 2 and exon 3 of GLIS2 by back-splicing. The divergent (red) and convergent (blue) primers were designed to identify the back-splicing structure of circGLIS2. **b** PCR analysis was performed to amplify the cDNA and gDNA of CRC cells, using the convergent (left) and divergent (right) primer, respectively. **c** Agarose gel electrophoresis (left) and qRT-PCR (right) were performed to detect circGLIS2 and linear GLIS2 mRNA after treatment with or without RNase R for 2 h. **d** circGLIS2 and GLIS2 mRNA levels were detected by qRT-PCR in DLD1 cells treated with actinomycin D (ACT-d) for 24 h. Data are represented as mean ± SD. **P < 0.01.

CircGLIS2, upregulated in CRC, is a novel circRNA generated from *GLIS2*. It consists of exons 2 and 3 of the GLIS2 gene. To verify the specific back-splicing sites, we designed divergent primers for specific back-splicing sites. In addition, convergent primers, which crossed exon 2 and 3 of GLIS2, were designed to work as normal control (Fig. 2a). We performed a set of PCR experiments to validate that circGLIS2 harbors a closed-loop structure that is not produced by trans-splicing or genomic rearrangement in CRC cells. First, the genomic DNA (gDNA) and transcriptomic complement DNA (cDNA) were obtained. We applied PCR for cDNA and gDNA using the divergent and convergent primers, respectively. The results showed that the convergent primers amplified GLIS2 from two kinds of DNA template. However, the divergent primers amplified the sequence that only existed in the cDNA template (Fig. 2b). Furthermore, RNase R treatment was used to validate the circular form of circGLIS2 because RNase R is an exonuclease that is incapable of cutting circRNAs. As shown in Fig. 2c, RNase R digestion cleared out most of the linear RNAs such as GLIS2 mRNA but circGLIS2 was more resistant. To verify the stability of circGLIS2, we used actinomycin D (ACT-d) to inhibit new RNA synthesis. As shown in Fig. 2d, circGLIS2 was more stable than the linear GLIS2 mRNA intracellularly. These data suggest that circGLIS2 is a circRNA that is generated from back-splicing of the *GLIS2* gene transcript.

### CircGLIS2 modulates CRC cell migration

**Fig. 3:**
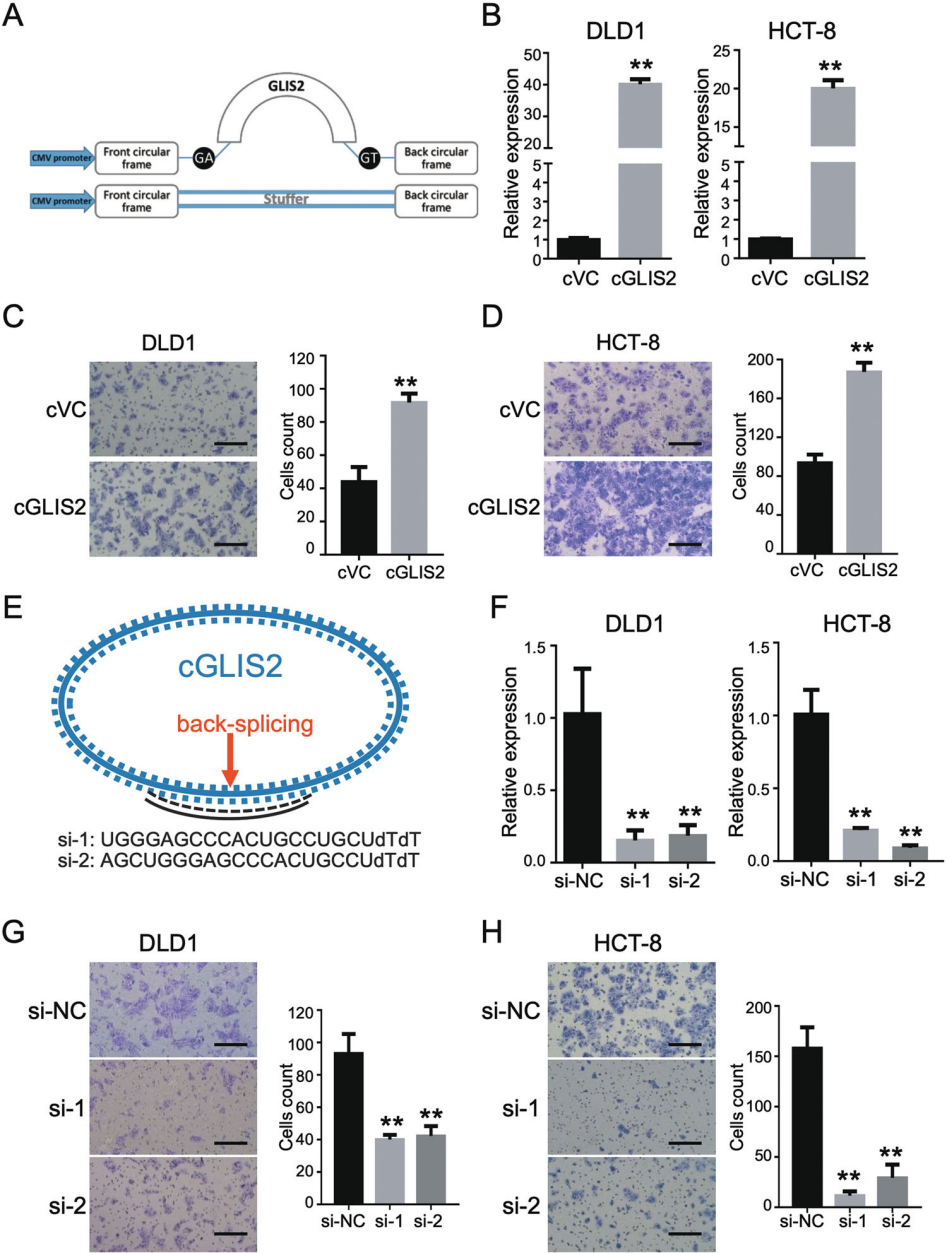
CircGLIS2 promotes CRC cell migration. **a** Schematic of circGLIS2 overexpression plasmid construction. **b** qRT-PCR validated circGLIS2 upregulation in DLD1 and HCT-8 cells transfected with circGLIS2 (cGLIS2). **c** Transwell assay showing the migration ability of DLD1 cells that overexpressed circGLIS2 (cGLIS2). **d** Transwell assay showing the migration ability of HCT-8 cells that overexpressed circGLIS2 (cGLIS2). **e** Schematic representation of the siRNA that targets the back-splicing junction of circGLIS2. **f** qRT-PCR assay was performed to validate the knockdown efficiency of the indicated siRNA in DLD1 and HCT-8. **g** Transwell assay showing the migration ability of DLD1 that were transfected with circGLIS2 targeting siRNA. **h** Transwell assay showing the migration ability of HCT-8 that were transfected with circGLIS2 targeting siRNA. Scale bars: 250 μm. Bars represent the number of migrated cells. Data are represented as mean ± SD. **P < 0.01.

To investigate the role of circGLIS2 in CRC, we cloned the circGLIS2 sequence into a circRNAs expression vector that consisted of two cyclization elements on the back-splicing site (Fig. 3a). The lentiviruses were generated using a second-generation lentivirus packaging system in which 293T cells were co-transfected with psPAX2, pMD2.G and the expression vector. DLD1 and HCT-8 were infected with the lentivirus and then screened with puromycin for a stably transfected cell line. First, we designed specific qPCR primers targeting the back-splicing site of circGLIS2 to detect circGLIS2 expression. The specificity of the primers was validated using a melting curve analysis of the qPCR-amplified product, which all showed a single peak (Fig. S1). Next, qPCR assays demonstrated that circGLIS2 was stably overexpressed in the DLD1 and HCT-8 CRC cell lines (Fig. 3b). Then, the circGLIS2 overexpressing cells were subjected to a transwell assay to test their cell migration properties. As shown in Fig. 3c, d, overexpression of cricGLIS2 promoted cell migration of both the DLD1 and HCT-8 CRC cell lines.

We also designed two siRNAs that specifically targeted the back-splicing site for a loss-of-function study (Fig. 3e). First, we verified the known efficiency of the siRNA via RT-qPCR. As shown in Fig. 3f, both si-1 and si-2 could reduce the expression of circGLIS2 in DLD1 and HCT-8 cell lines. Then, as we expected, the downregulation of circGLIS2 inhibited cell migration in the DLD1 and HCT-8 cell lines (Fig. 3g, h). These data indicate that circGLIS2 is involved in the regulation of CRC cell migration.

### NF-κB signaling pathway is involved in circGLIS2 regulated cell migration

**Fig. 4:**
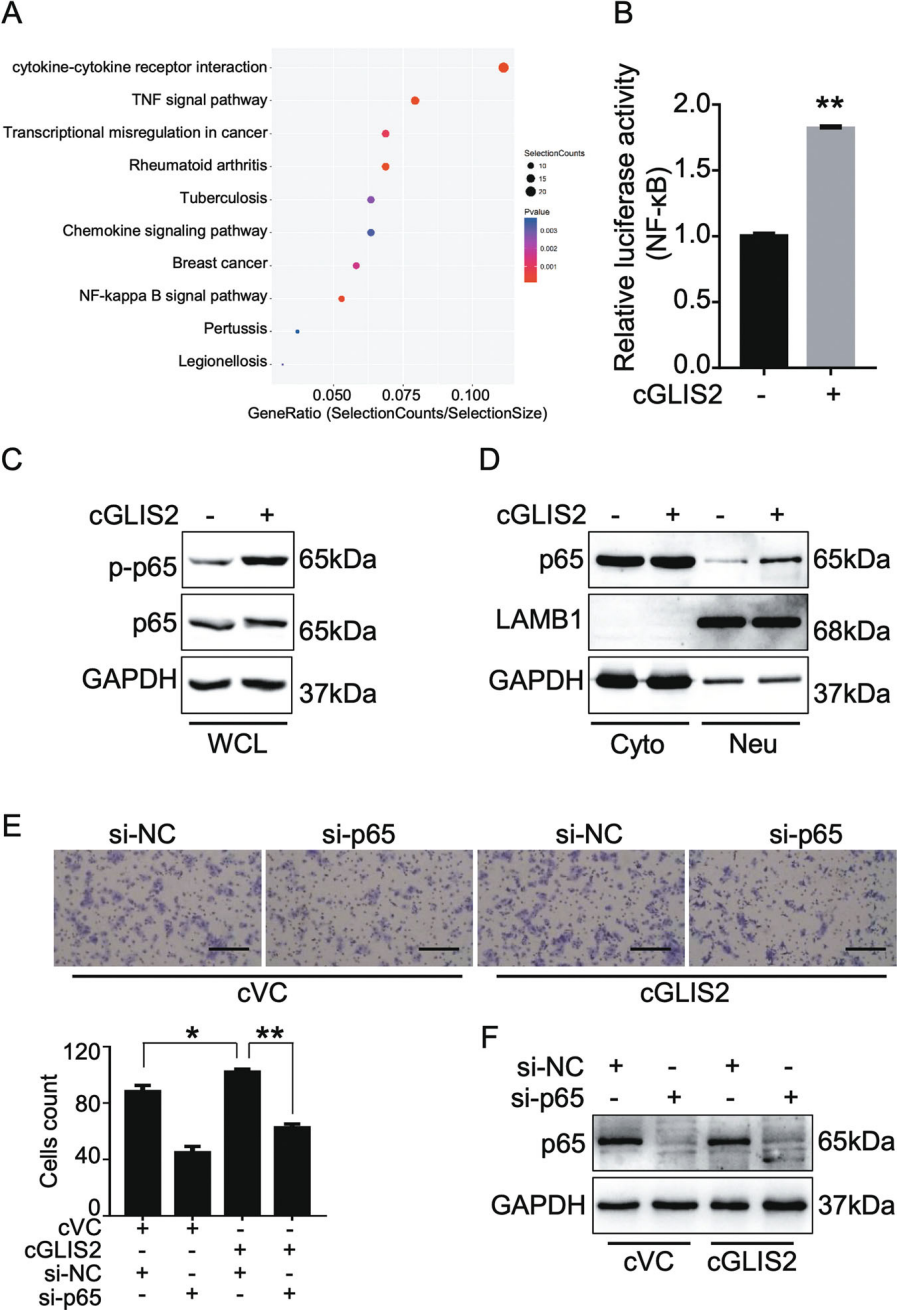
CircGLIS2 activates the NF-κB signaling pathway in colorectal cancer cells. **a** The top 10 significantly enriched KEGG pathways of the differentially expressed genes in DLD1/cGLIS2 cells compared to DLD1/VC cells. The y-axis shows the name of the pathway, and the x-axis shows the enrichment factor. **b** Luciferase reporter assays were performed to detect NF-κB signaling pathway activity in DLD1/cGLIS2 cells compared with DLD1/VC cells. **c** Whole cell lysis (WCL) western blot assay was used to detect the levels of p65 and p-p65 after circGLIS2 overexpression in DLD1 cells. **d** Cytoplasm and nuclei protein separation checked with a Western blot assay in order to detect the p65 level in nuclei. LAMB1 and GAPDH were used as nuclear and cytoplasm markers, respectively. **e** Transwell assay illustrated the migration ability of DLD1/VC (cVC) and DLD1/cGLIS2 (cGLIS2) cells after knockdown of p65 via siRNA transfection. Scale bars: 250 μm. Bars represent the number of migrated cells. Data are represented as mean ± SD. *P < 0.05, **P < 0.01. **f** Western blot assays were performed to validate the knockdown efficiency of the siRNA targeted p65.

To explore the basic mechanism underlying the effect of circGLIS2 on cell migration, we screened the signaling pathways that may participate in circGLIS2 functions using the Arraystar Human LncRNA Microarray V4.0 and KEGG analysis of the cricGLIS2 overexpressed DLD1 cell line. The data of the microarray profiling have been deposited in Mendeley Data (DOI: 10.17632/fms7h9xwby.1). In contrast to the control cells, 545 genes were upregulated and 166 genes were downregulated in the circGLIS2 overexpressed cells (Fold Change cut-off: 2.0). KEGG pathway enrichment analysis indicated that the differently expressed genes induced by circGLIS2 overexpression were largely involved in the TNFα and the NF-κB signaling pathway (Fig. 4a). Accordingly, we conducted the luciferase vector pGL4.32 containing five copies of an NF-κB response element (NF-κB-RE), which drives expression of the luciferase reporter gene luc2P (Photinus pyralis) to detect the activity of NF-κB pathway in cells with circGLIS2 overexpression. As shown in Fig. 4b, NF-κB signal was activated after the transfection of overexpressed circGLIS2 in DLD1 cells. Given that p65 plays a crucial role in the NF-κB signaling pathway, we also investigated whether circGLIS2 could induce p65 activity. Western blots showed that the level of phosphorylated p65 (p-p65) had increased following the overexpression of circGLIS2 (Fig. 4c) and that circGLIS2 overexpression increased the nuclear localization of p65 (Fig. 4d). To further confirm the relationship between circGLIS2 and NF-κB signaling pathway, p65 targeted siRNA was applied to knockdown the level of p65 (Fig. 4f). Accordingly, the pro-migration effect was eliminated under the p65 knockdown circumstance (Fig. 4e). These data suggest that circGLIS2 is a positive regulator of the NF-κB signaling pathway.

### CircGLIS2 enhances NF-κB signaling pathway and cell motility potential of CRC cells by an autocrine mechanism

**Fig. 5:**
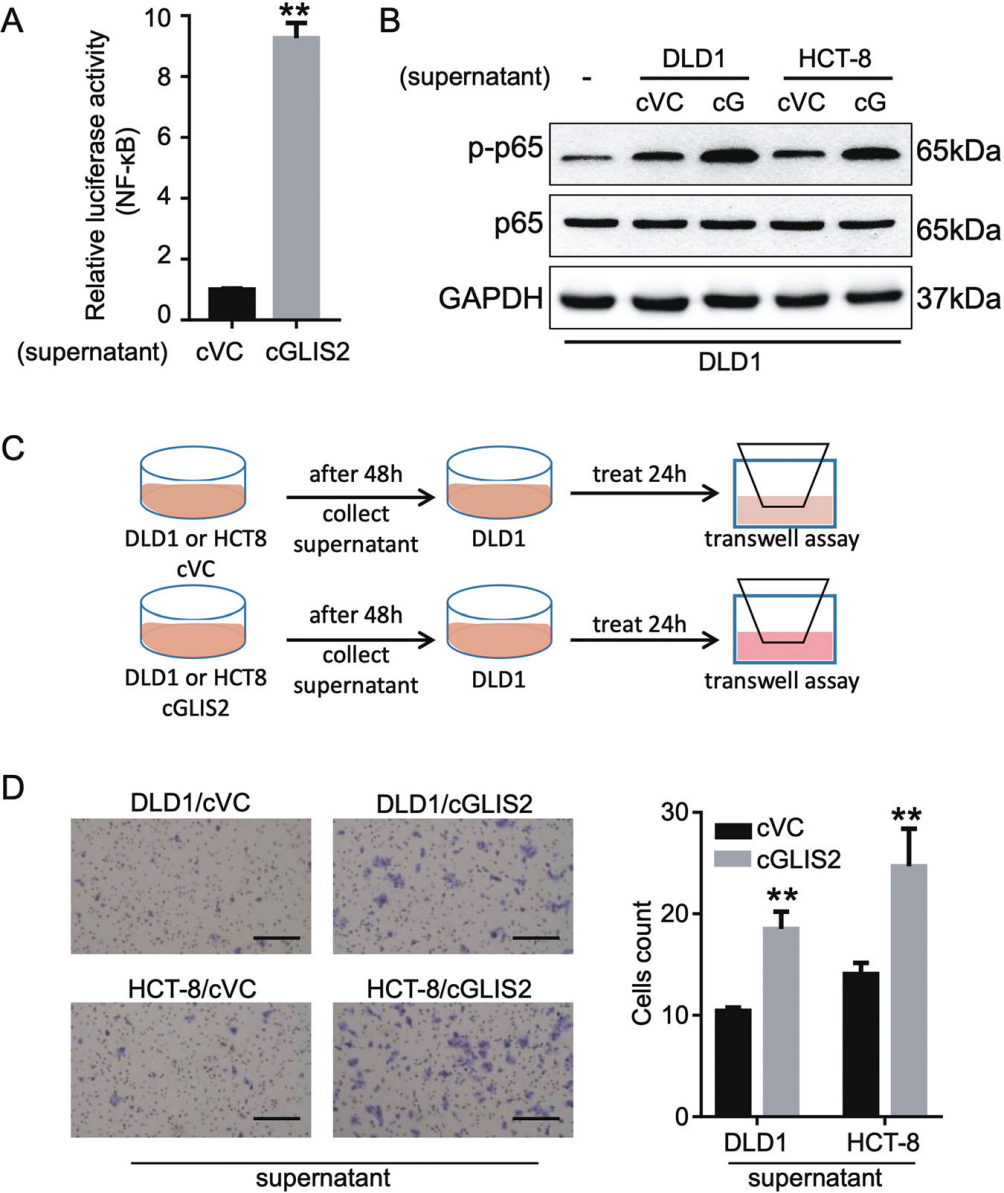
CircGLIS2 overexpressed cancer cell-derived supernatant induces NF-κB signaling and motility activation. **a** Luciferase report assay of pGL4.32 transfected 293 T cells co-culture with the supernatant of DLD1 cells (cVC or cGLIS2). **b** Western blot assay showing the p-p65 and p65 levels in DLD1 cells co-culture with the supernatant of circGLIS2 overexpression (cG) or vector control (cVC) CRC cells. **c** Schematic protocol for the supernatant associated transwell assay. **d** Transwell assay showing the migration ability of DLD1 that were co-culture with the supernatant of circGLIS2 overexpression or vector control CRC cells. Scale bars: 250 μm. Bars represent the number of migrated cells. Data are represented as mean ± SD. **P < 0.01.

The above data suggest that circGLIS2 was a potential regulator of NF-κB signal pathway, which plays an important role in the tumor associated microenvironment (TME). We hypothesize that circGLIS2 overexpressing cancer cells manipulate cancer development via TME construction. To test this hypothesis, we first transfected the vector pGL4.32 into 293T cells to detect the effect of cell supernatant on NF-κB signal pathway. Supernatants of circGLIS2 overexpression cells and the vector control cells were collected as condition medium. We found that the activity of luciferase in NF-κB was significantly increased by the circGLIS2 overexpression condition medium (Fig. 5a). Additionally, we also confirmed that the p65 phosphorylation level was elevated in CRC cells when the cells were treated with circGLIS2 overexpression condition medium (Fig. 5b). To further investigate the interaction between circGLIS2 associated microenvironment and cancer cell motility. We designed an in vitro model for measuring cell migration after the condition medium treatment (Fig. 5c). CRC cells were pretreated with the condition medium for 24 h and then measure their cell motility by the transwell assay. We found that circGLIS2 overexpression condition medium endowed CRC cells with greater motility (Fig. 5d). Collectively, our data suggested that the supernatant of circGLIS2 overexpressing cells was sufficient to enable the NF-κB signal pathway activation and cell motility promotion.

### CircGLIS2-overexpressed CRC cells amplify the chemokines milieu to recruit neutrophils via autocrine and paracrine manners

**Fig. 6:**
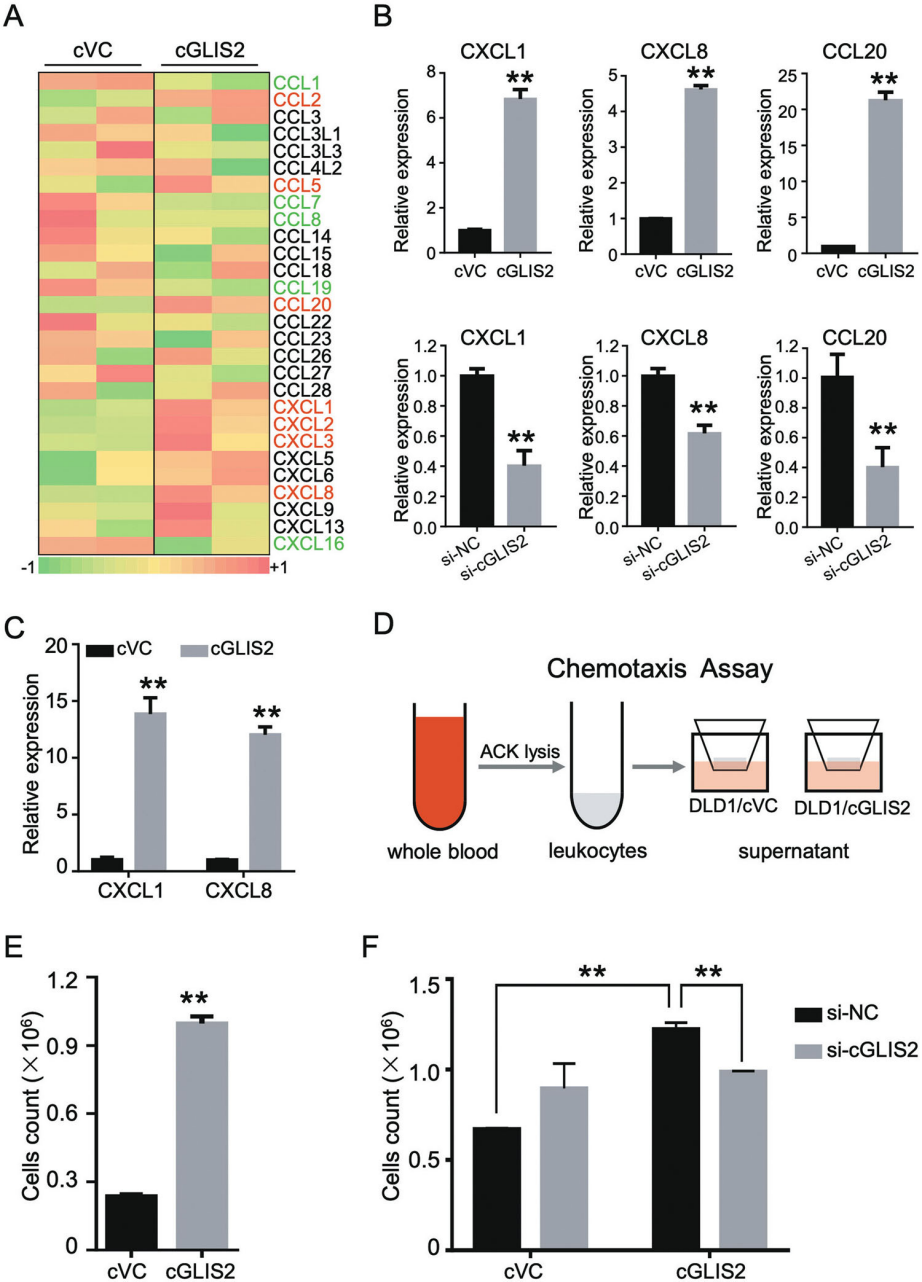
CircGLIS2 overexpressed cancer cells construct chemokines milieu to recruit neutrophils from peripheral blood. **a** A heatmap showing chemokines expression in circGLIS2 overexpressed and vector control DLD1 cells (*n* = 2). Upregulated chemokines were marked in red and downregulated chemokines were in green. **b** qRT-PCR analysis of CXCL1, CXCL8 and CCL20 expression in circGLIS2 overexpressed (up) or silenced cells (down). **c** qRT-PCR analysis of CXCL1 and CXCL8 expression in DLD1 cells co-cultured with the supernatant of circGLIS2 overexpression or vector control CRC cells. **d** Schematic protocol for the in vitro chemotaxis assay of circGLIS2-associated supernatant. **e** Transwell assay analysis of chemotactic activity of leukocytes toward the supernatant of DLD1 cells (cVC or cGLIS2). **f** Transwell assay was used evaluated the chemotactic activity of leukocytes toward the supernatant of DLD1/cVC and DLD1/cGLIS2 cells transfected with si-NC or si-cGLIS2. Data are represented as mean ± SD. **P < 0.01.

To identify what factors contribute to the tumor microenvironment, we further analyzed the specific gene set of differentially expressed genes caused by overexpression of circGLIS2 in DLD1 cells (Fig. 6a). It is worth noting that some chemokines (CXCL1, CXCL8, and CCL20) were elevated in DLD1-circGLIS2 cells compared to the vector control cells, which can be confirmed through knocking-down circGLIS2 (Fig. 6b). We next investigated whether the condition medium from circGLIS2 overexpression cells could also induce the expression of CXCL1 and CXCL8 in CRC cells. As shown in Fig. 6c, when CRC cells were exposed in the condition medium of circGLIS2-overexpressing cells, the expression of CXCL1 and CXCL8 were significantly increased. This data indicated that circGLIS2-overexpressing cells amplified the chemokines milieu within the TME via autocrine manner. CXCL1 and CXCL8 are known chemokines for neutrophils. Thus, we speculated that circGLIS2 overexpression cells could recruit leukocyte via the construction of tumor associated microenvironment. We designed an in vitro chemotaxis assay to evaluate the intercellular role of the circGLIS2 associated microenvironment (Fig. 6d). We collect the leukocytes from healthy donors via the ACK lysis process. The leukocytes were added to the upper chamber, while the supernatant of cricGLIS2 overexpression cell or vector control cell was add to the lower chamber. Surprisingly, the conditioned medium of circGLIS2 overexpressed cells recruit more cells to the lower chamber (Fig. 6e). Also, we identified the properties of leukocytes in the upper and lower chamber via flow cytometry and we found that the supernatant of circGLIS2 overexpressed cell attracted more CD15^+^ neutrophils in the lower chamber (Fig. S2). Moreover, silencing circGLIS2 suppressed the chemotaxis of leukocytes toward cell supernatant (Fig. 6f). These results suggest that cricGLIS2 cells form a unique tumor microenvironment enriched with neutrophils via autocrine and paracrine manners.

### CircGLIS2 binds to miR-671 to activate the NF-κB signaling pathway

**Fig. 7:**
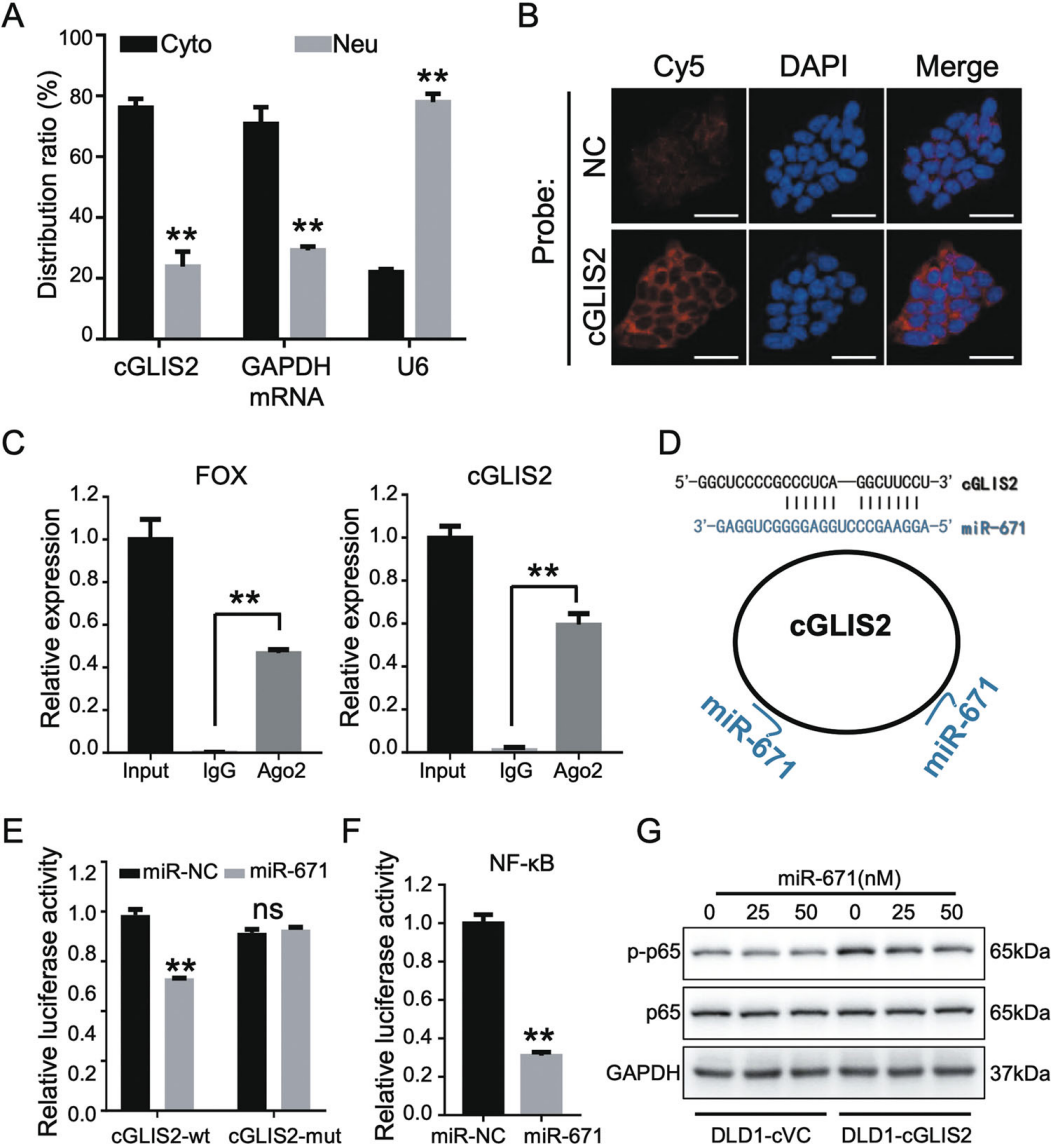
CircGLIS2 binds to miR-671 to modulate the NF-κB signaling pathway. **a** Subcellular distribution of circGLIS2 was detected by qRT-PCR, GAPDH was used as the cytoplasm-located (Cyto) control and U6 was used as the nucleus-located (Neu) control. **b** FISH illustrated that circGLIS2 was predominantly located in the cytoplasm. Oligo DNA probes target circGLIS2 was labeled with Cy5 (red) and the nuclei was stained with DAPI (blue). Scale bars: 25 μm. **c** RIP-IP assays were performed to co-immunoprecipitation the Ago2 complexes from DLD1 cells. qRT-PCR was used to detect the circGLIS2 level and FOX was used as the positive control. **d** A schematic drawing showing the putative binding sites of miR-671 on circGLIS2. **e** The luciferase activity of wild-type circGLIS2 or mutant circGLIS2 after transfection with miR-671 mimic or miR-NC. **f** NF-κB signaling pathway activity was assessed in cells transfected with miR-671 or miR-NC. **g** Western blot assay showing the p-p65 and p65 levels in DLD1/cVC and DLD1/cGLIS2 cells transfected with different amounts of miR-671 or miR-NC. Data are represented as mean ± SD. **P < 0.01.

To further explore the molecular mechanism by which circGLIS2 activates the NF-κB signaling pathway, we first determined the subcellular location of circGLIS2 in CRC cells. qPCR analysis of the subcellular fractionated RNA showed that circGLIS2 predominantly resided in the cytoplasm of CRC cells (Fig. 7a). FISH experiments also showed the same results (Fig. 7b). There are many possible mechanisms of cytoplasmic circRNAs that have been reported, including sponging miRNAs and interacting with other RNA binding proteins. We analyzed the protein binding function of circGLIS2 using the CircInteractome database^13^. The results indicated that circGLIS2 probably interacts with AGO2, which is a very common miRNA interaction protein. Along this line, a RAP assay showed that an anti-AGO2 antibody did enrich circGLIS2 (Fig. 7c). Therefore, we speculated that circGLIS2 might function by interacting with miRNAs.

**Fig. 8:**
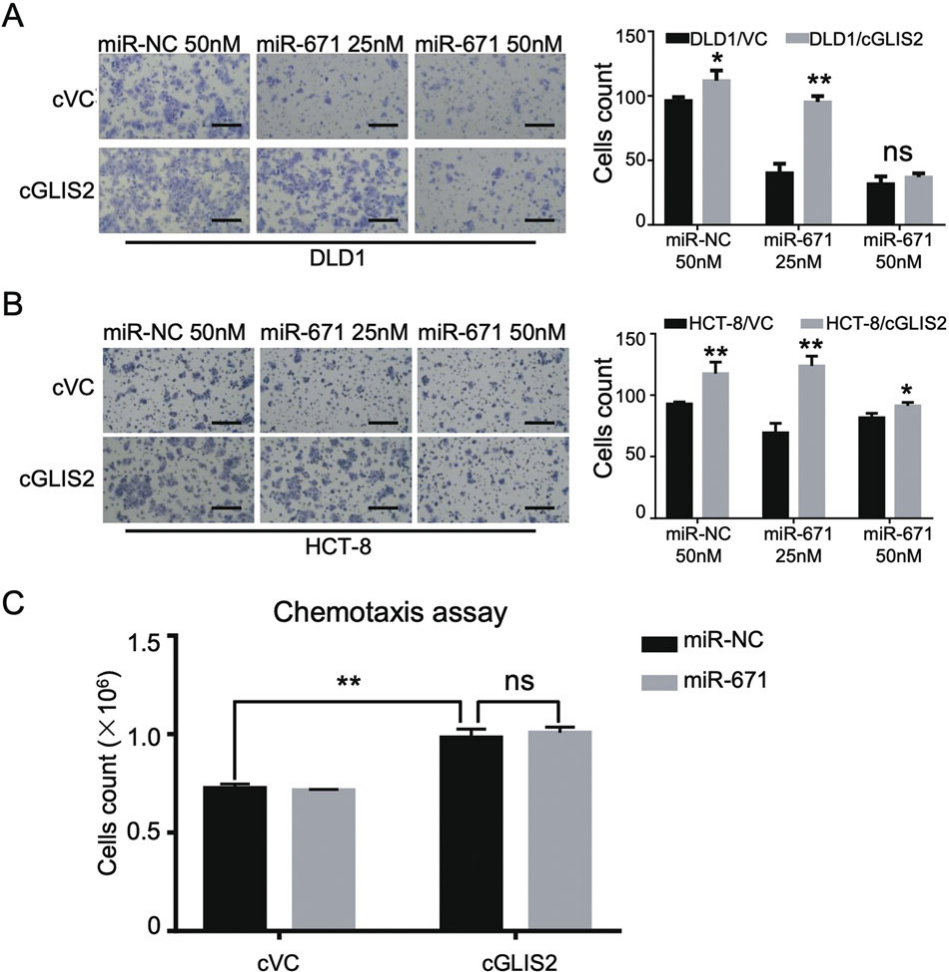
MiR-671 attenuates the function of circGLIS2 in cell migration but not affecting chemotaxis potential. **a** Cell migration activity was assessed for DLD1/VC and DLD1/cGLIS2 cells that were transfected with different amounts of miR-671 or miR-NC. **b** Cell migration activity was assessed for HCT-8/VC and HCT-8/cGLIS2 cells that were transfected with different amounts of miR-671 or miR-NC. Scale bars: 250 μm. **c** Transwell assay was used to evaluate the chemotactic activity of leukocytes toward the supernatant of DLD1/cVC and DLD1/cGLIS2 cells transfected with miR-NC or miR-671. Data are represented as mean ± SD. *P < 0.05, **P < 0.01.

To validate this hypothesis, the CircInteractome bioinformatics databases were used to predict miRNAs that may bind to circGLIS2^13^. Based on the number of pairing sequence and TargetScan miRNA perdition score, three miRNAs were selected for transwell assay validation. Among the candidate miRNAs, miR-671 significantly inhibited cell migration in DLD1 cells (Fig. S4). To detect miR-671 expression in CRC tissue, we referred to TCGA dataset and revealed that miR-671 was downregulated in colon and rectum adenocarcinoma (Fig. S3). Also, miR-671 has been reported to regulate cancer progression and immunomodulation^14^. Thus, we hypothesized that circGLIS2 might regulate the NF-κB signaling pathway by competitively binding to miR-671 (Fig. 7d). We used a dual-luciferase reporter system to validate the interaction between circGLIS2 and miR-671. First, we generated wild-type and mutated vectors of circGLIS2 validated by Sanger sequencing. Then, the wild-type or mutated circGLIS2 vectors were co-transfected with miR-671 into the DLD1 cell line. The dual-luciferase reporter assays indicated that the relative luciferase activity had decreased after co-transfection with circGLIS2-WT and miR-671 (Fig. 7e). We evaluated the activity of the NF-κB pathway in cells after transfection with miR-671 and found that miR-671 suppressed NF-κB signaling in DLD1 cells (Fig. 7f). To investigate whether circGLIS2 exerts its function via miR-671, rescue experiments were performed with circGLIS2 ectopic expressing cells with the presence of miR-671 dosage transfection. MiR-671 reversed the ability of circGLIS2 to up-regulate p-p65 in CRC cells (Fig. 7g). Also, miR-671 reversed the ability of circGLIS2 to promote the migration of DLD1 and HCT-8 cells (Fig. 8a, b). However, we did not observe a significant reversal effect in the cell chemotaxis experiments (Fig. 8c). These findings demonstrated that circGLIS2 could bind to miR-671 to increase the activity of the NF-κB signaling pathway and facilitate its own migration.

## Discussion

circRNAs have long been recognized as a byproduct of gene expression in mammalian cells^15^. Recently, with the development of RNA-seq and bioinformatics technologies, many circRNAs have been found to have specific roles in various diseases, including cancer^16^. It has been reported that, circMAN2B2, circPRKCI, circPIP5K1A, and circPVT1 can promote tumor growth and invasion in lung cancer^17–20^. In addition, circBANP can increase the metastatic phenotype in CRC^21^, and circ-102004 can promote cancer cell proliferation in prostate cancer^22^. Furthermore, circ-0013958 might be used as a potential biomarker in liquid biopsies for lung adenocarcinoma^23^. CircPAN3 is enriched in refractory acute myeloid leukemia and might serve as a chemo-sensitive predictor for the patients^24^. Thus, it is essential to analyze the differentially expressed circRNAs in CRC and elucidate its basic mechanism in cancer progression.

Here, by combining circRNAs array analysis with GEO database verification, we found that circGLIS2 was upregulated in CRC. CircGLIS2 is located on the plus strand of chromosome 16p13.3 and is derived from the known protein-coding gene GLIS2. As the host gene of circGLIS2, GLIS2 plays the role of a transcription factor and may act as an oncogene or tumor suppressor depending on the cell circumstances. Previous reports showed that GLIS2 is usually involved in the development of cancer as fusion genes. For example, GLIS2 fused with CBFA2T3 directly contributes to leukemogenesis and presents an aggressive phenotype^25^. ETO-GLIS2 fusion gene oligomerization promotes megakaryocytic differentiation^26^. These facts suggested that GLIS2 has various gene structures and functions in the genome. However, the circRNAs derived from GLIS2 as well as their function in cancer progression are still poorly understood. Here, the gain-and-loss function assays showed that consistent expression or silencing of the expression of circGLIS2 in CRC cell lines significantly promoted or inhibited cell migration, respectively. These data suggest that circGLIS2 is involved in the progression of CRC via modulating the migration phenotype of the cancer cells. Similar to other noncoding RNAs, circRNAs are involved in cellular gene regulation through post-transcriptional modification^27,28^. Emerging research has indicated that circRNAs function as “miRNAs sponges” to competitively bind to miRNAs^4^. Subsequently, our further data showed that circGLIS2 settled in the cytoplasm and was incorporated into the AGO-RISC complex in CRC cells. Our study focused on the interaction between circGLIS2 and miR-671 in CRC. We found that miR-671 inhibited the NF-κB signaling pathway in CRC cells. Also, miR-671 attenuated NF-κB signaling activity that was elicited by circGLIS2 overexpression. In the rescue experiments, we observed a gradual inhibition of cell motility with miR-671 transfection in DLD1 and HCT-8. Moreover, we revealed that miR-671 was significantly downregulated in CRC tissue compared to the normal tissue. It has been known that constitutive activation of NF-κB signaling pathway plays a role in the progression of cancers^29^. Previous reports showed that abnormally activated NF-κB signaling was found in 40% of CRC patients. TRAF6 activates NF-κB via the nuclear shuttling effect of p65 in CRC^30^. RNA helicase p68 enhanced NF-κB target genes expression by directly promoting p65 transcription^31^. Our research introduced a new mechanism that helps understand the NF-κB pathway in CRC. We found that circGLIS2 activated NF-κB pathway through competitively binding to miR-671. However, we also noted that circGLIS2 could interact with other miRNAs including miR-512, miR-874 and miR-940, since these miRNAs also have targeting sequences and high scores in bioinformatics prediction. We also cannot rule out whether circGLIS2 would function by binding to proteins or translating peptides. The involvement of miR-671 clarified the role of circGLIS2 in NF-κB and cell migration, but miR-671 does not affect the leukocyte recruitment. This question awaits further investigation.

Our results showed that more neutrophils were recruited when the leukocytes were exposed to the supernatant of circGLIS2 overexpressed cells. Recent studies have highlighted an important role of neutrophils in manipulating cancer metastasis^32^. In primary cancers, infiltrating neutrophils promote cancer cell dissociation by secreting TGF-β and MMP9^33,34^. In the circulating cancer cells (CTC), neutrophils physically bind with circulating cancer cells to promote intravasation of CTC^35^. Notably, an increase in the ratio of neutrophil-to-lymphocyte has been observed in many kinds of cancers and has become a poor survival indicator in cancer patients^36^. This indicated that circGLIS2 might induce a pro-metastasis microenvironment to promote metastasis of cancer cells.

In summary, all of the data indicate that the upregulated circGLIS2 contributes to pro-metastasis microenvironment via activation of NF-κB signaling pathway and recruitment of leukocytes in CRC. Mechanically, circGLIS2 acts as a “miRNAs sponge” to inhibit miR-671 function and subsequently manipulate the NF-κB signaling axis. These findings highlight a new mechanistic connection between NF-κB and circGLIS2 in the construction of a pro-metastasis microenvironment of CRC. These results may provide new insights and lead to new therapeutic strategies for CRC prevention and treatment.

## Materials and methods

### Tissue samples and ethics statement

Three pairs of CRC tissues and matched adjacent noncancerous colorectal tissues were collected from surgical resections at the Sixth Affiliated Hospital, Sun Yat-sen University (Guangdong, China). None of the tissues were exposed to chemo- or radio-therapies prior to surgery. The tissues were frozen and stored in liquid nitrogen until use. Whole blood samples were preserved at 4 °C after collection from healthy donor. The leukocyte were purified by ACK red blood cells depletion kit (Leagene, Beijing, China), according to the manufacturer’s manual. The protocol and the use of clinical materials for research purposes were approved by the Ethics Committee of the Sixth Affiliated Hospital of Sun Yat-sen University (ethical approval number 2018ZSLYEC-008).

### Cell culture and transfection

The normal human colon epithelial cell line NCM460 was maintained in our lab and the CRC cell lines DLD1, HCT-8, HCT116, RKO, HT-29 and HCT-15 were obtained from the American Type Culture Collection (ATCC, Virginia, USA). The NCM460 cell line was cultured in DMEM (Gibco, New York, USA) supplemented with 10% fetal bovine serum (FBS, Gibco, New York, USA) and 100 U/mL penicillin-streptomycin (Gibco, New York, USA). The other CRC cell lines (DLD1, HCT-8, HCT116, RKO, HT-29, and HCT-15) and their corresponding construction cell lines were cultured in RPMI1640 (Gibco, New York, USA) supplemented with 10% FBS and 100 U/mL penicillin-streptomycin. All cells were cultured in a humidified atmosphere with 5% CO_2_ at 37 °C. Cells were passaged every 2–3 days to maintain cell viability.

### Microarray analysis

For circRNA expression profiling, Arraystar Human circRNA Array v2 analysis was performed by Kangcheng Biotech (Shanghai, China) according to Arraystar standard protocols. Briefly, total RNAs were extracted from the paired tissue samples of three CRC patients by TRIzol reagent (Invitrogen, Oregon, USA). Then the RNAs were digested with RNase R (Epicentre, Wisconsin, USA) to remove the linear RNA population. The remained RNAs were amplified and transcribed into fluorescent cRNA and then hybridized onto the Arraystar Human circRNA Array (8 × 15 K, Arraystar). After washing the slides, the arrays were scanned by the Agilent Scanner G2505C. Agilent Feature Extraction software was used to analyze acquired array images. Quantile normalization and subsequent data processing were performed using the R software package. Volcano Plot filtering was used to identify the statistical significance of differentially expressed circRNAs between two groups.

For mRNA expression profiling, Arraystar Human LncRNA Microarray V4.0 was used to analyze protein-coding transcripts by Kangcheng Biotech (Shanghai, China). Briefly, mRNA was purified from total RNA after removal of rRNA (mRNA-ONLY™ Eukaryotic mRNA Isolation Kit, Epicentre). Then, the RNA sample was transcribed into fluorescent cRNA (Arraystar Flash RNA Labeling Kit, Arraystar). The labeled cRNAs were purified by RNeasy Mini Kit (Qiagen) and then hybridized onto the LncRNA expression microarray slide. The hybridized arrays were washed, fixed and scanned by Agilent DNA Microarray Scanner G2505C. Agilent Feature Extraction software (version 11.0.1.1) was used to analyze acquired array images. Quantile normalization and subsequent data processing were performed using the GeneSpring GX v12.1 software package (Agilent Technologies). Differentially expressed mRNAs were identified through P-value/FDR and Fold Change filtering.

### RNA extraction and quantitative real-time polymerase chain reaction (qRT-PCR)

Total RNA was extracted using TRIzol reagent (Invitrogen, Oregon, USA), reverse transcription was performed using PrimeScript RT Master Mix (Takara Biomedical Technology, Beijing, China), and quantitative real-time PRC was performed using SYBR Green Premix Ex Taq II (Takara Biomedical Technology, Beijing, China) according to the manufacturer’s instructions. The results of the transcript levels were analyzed by the 2-delta-delta Ct method. All quantitative real-time PCR primers were synthesized in Sango Biotech (Shanghai, China) and the primers sequence shown as follows: circGLIS2-forward, CAGCAGCTCGCTGTCCCCCGAGCG; circGLIS2-reverse, GTTGGAGGTGGCAGCAGGCAGTGG. GAPDH-forward, GCACCGTCAAGGCTGAGAAC; GAPDH-reverse, TGGTGAAGACGCCAGTGGA. U6-forward, CTCGCTTCGGCAGCACA; U6-reverse, AACGCTTCACGAATTTGCGT. And the divergent and convergent primer set for circGLIS2 PCR identification were shown as below: divergent primer pair, CTTCCTGCTGAACTCCAAGT and GCTTGGTGATACTCAGCTTC; convergent primer pair, TTCCTGCGTGAAGACCAGCT and GAACTTGGAGTTCAGCAGGA.

### Cell migration assays

The cell migration activity was assessed using 24-well transwell insert chamber plates (Corning, Massachusetts, USA). Briefly, Cells were harvested then suspended in serum-free 1640 medium. Then cells were transferred in to the upper compartment of the chamber at 0.2 million in 200 μL. A total of 600 μL of complete 1640 medium supplemented with 10% FBS was added to the lower chamber. The cells were then incubated for 24 h. The cells that did not migrate were scraped off with a cotton swab and the cells were stained with a crystal violet solution (Beyotime, Jiangsu, China). The cells were then counted and analyzed. These experiments were repeated three times.

### Western blot

Cells were harvested and lysed with RIPA buffer (Beyotime, Jiangsu, China) followed by BCA analysis to determine the protein concentration. The samples were separated by SDS-PAGE and transferred onto nitrocellulose membranes. After incubation with the primary antibodies anti-p65 (1:1000; AF0246, Beyotime, Jiangsu, China), anti-phosphor p65 (1:1000; AN371, Beyotime, Jiangsu, China), anti-GAPDH (1:10000; 10494–1-AP, Proteintech Group, Illinois, USA), anti-LAMB1 (1:5000; 23498–1-AP, Proteintech Group, Illinois, USA), the membranes were then incubated with a secondary antibody (1:5000; #7074, Cell Signaling Technology, Massachusetts, USA). After washing, signals were detected using a chemiluminescence system (Bio-Rad, California, USA) and analyzed using Image Lab Software (Bio-Rad, California, USA).

### Chemotaxis assay

Leukocytes were isolated from whole blood using ACK red blood cells depletion kit (Leagene, Beijing, China), according to the manufacturer’s protocol. Purified leukocytes were counted and 2 × 10^5^ cells were added to the upper chamber. The supernatant of DLD1/VC or DLD1/circGLIS2 cells was added to the lower chamber to test the chemotaxis effect. After 2 h of incubation, the number of leukocytes translocated to the lower chamber were counted using The Bio-Rad TC20 automated cell counter (Bio-Rad, California, USA). Subsequently, leukocytes obtained from the lower chamber were labeled with FITC conjugated CD3 (Biolegend, California, USA) and APC conjugated CD15 (Biolegend, California, USA) antibodies to test the identity of the leukocytes recruited by the supernatant of CRC cells. The samples were analyzed on FACSCanto Flow Cytometry (BD Biosciences, California, USA) according to the user’s manual.

### RISC Ago2 immunoprecipitation

RISC immunoprecipitation assays were performed with an Ago2-RIP Kit (Millipore, Massachusetts, USA) as directed by the manufacturer. Briefly, DLD1-circGLIS2 cells were cross-linked through 16% formaldehyde treatment and lysed in co-IP lysis buffer containing proteinase inhibitors and RNase inhibitor. The lysate was incubated with anti-Ago2 antibody overnight. Agarose beads were added to the co-IP sample to precipitate the RISC-Ago2 complex. The beads were washed with co-IP lysis buffer six times. The RNA was purified using TRIzol reagent (Invitrogen, Oregon, USA) according to the manufacturer’s instructions.

### RNase R treatment

Total RNA was extracted using TRIzol reagent according to the manufacturer’s instructions. The RNA was incubated with 20 U RNase R at 37 °C for 2 h in the buffer provided with the kit (Epicentre, Wisconsin, USA).

### Luciferase reporter assay

Cells were co-transfected with plasmids containing the targeted sequences of the wild or mutant fragment from the circGLIS2 and miRNAs mimics using Lipofectamine3000 (Invitrogen, Oregon, USA) according to the manufacturer’s instructions. Then, 24 h after transfection, firefly luciferase activities were measured by using a dual-luciferase reporter assay kit (Promega, Massachusetts, USA), with renilla activity used as the internal control. Finally, the ratios of firefly to renilla luminescence represent the binding activity of circGLIS2 and miRNAs.

For the NF-κB signaling pathway report system, cells were transfected with the pGL4.32/NF-κB reporter plasmid (Promega, Massachusetts, USA) for 24 h, and firefly luciferase activity was then measured by using a luciferase reporter assay kit (Promega, Massachusetts, USA). The luminescence was normalized against the protein concentration.

### Fluorescence in situ hybridization (FISH)

Specific DNA probes to the circGLIS2 back-splicing site were applied for in situ hybridization. In brief, cells were seeded onto a coverslip following by fixation with 25% formamide (Sigma-Aldrich, Missouri, USA). The protein fraction was removed by 20 µg/mL proteinase K (Sigma-Aldrich, Missouri, USA) treatment for 10 min. The sample was then dehydrated in a gradient of ethanol solutions. Before the hybridization, the Cy5-labeled probes (Sangon Biotech, Shanghai, China) were diluted in hybridization buffer at 10 ng/mL. Following a 95 °C denaturing process, they were chilled on ice. The sample was covered with the denatured probes and incubated in a 60 °C incubator overnight. The coverslip was gently washed with 2× SSC buffer (Leagene, Beijing, China) 5 times. The nuclei were stained with Hoechst 33342 (Invitrogen, Oregon, USA). The fluorescent images were acquired on a Leica TCS SP8 DLS confocal microscope system (Leica, Hesse, Germany). The Cy5signal excited in response to the 649 nm laser and had an emission wave at 666 nm. The Hoechst 33342 signal was excited by a 346 nm laser and had an emission wave at 460 nm. FISH probes are as follows: FISH-circGLIS2 Cy5-TGCCTCCAGTGCCCAGTGCCTCGGTTCCTGCGTGAAGACCAGCTGG; FISH-NC Cy5- TGGTGTCCCCTGGACGTGGCTACACGTAGCACTCGGACGTGGCTA.

### Statistical analysis

All statistical analyses were carried out using GraphPad Prism (GraphPad Software, California, USA). Quantitative values are expressed as means ± standard deviation (SD) of at least three independent repetitions. The difference between means was evaluated by unpaired two-tailed Student’s *t* test. A probability value of *P* < 0.05 was used as the criterion for statistical significance.

## Supplementary information

Supplementary figures legends

Table S1

figure S1

figure S2

figure S3

figure S4
