# Supplementary material for: Circular RNA GLIS2 promotes colorectal cancer cell motility via activation of the NF-κB pathway
Source: Cell Death Dis. 2020 Sep 23;11(9):788. doi: 10.1038/s41419-020-02989-7 (PMC7511409; doi:10.1038/s41419-020-02989-7)
Supplement: Supplementary file 1 — Supplementary figures legends [file 41419_2020_2989_MOESM1_ESM.docx]

**Supplementary Figure Legends**

Fig. S1 Melting curve of circGLIS2 real-time quantitative PCR.
(a) Melting curve analysis of circGLIS2 in DLD1 cell line. (b) Melting curve analysis of circGLIS2 in HCT-8 cell line.

Fig. S2 Qualitative analysis of leukocytes in cell chemotaxis assay.
Flow cytometry analyzed the leukocytes from the upper and lower chambers that were stained with CD3 and CD15 markers. (b) The proportion of CD15+ neutrophil in the up or down chamber was compared between circGLIS2 overexpression cells (cGLIS2) and vector control cells (cVC). Data are represented as mean ± SD. *P < 0.05, **P < 0.01.

Fig. S3 The level of miR-671 was down-regulated in CRC.
(a) Expression profiling of miR-671 in The Cancer Genome Atlas Colon Adenocarcinoma (COAD) dataset. (b) Expression profiling of miR-671 in The Cancer Genome Atlas Rectum Adenocarcinoma (READ) dataset. Data are represented as mean ± SD. **P < 0.01.

Fig. S4 Transwell assay of cGLIS2-targeted miRNAs.
Transwell assay showing the migration ability of DLD1 cells that were transfected with cGLIS2-targeted miRNAs, including miR-512, miR-671 and miR-874. Scale bars: 250 μm. Bars represent the number of migrated cells. Data are represented as mean ± SD. **P < 0.01.
