# Supplementary material for: Circular RNA GLIS2 promotes colorectal cancer cell motility via activation of the NF-κB pathway
Source: Cell Death Dis. 2020 Sep 23;11(9):788. doi: 10.1038/s41419-020-02989-7 (PMC7511409; doi:10.1038/s41419-020-02989-7)
Supplement: Supplementary file 2 — Table S1 [file 41419_2020_2989_MOESM2_ESM.docx]

Table S1 Clinicopathological characteristics of the CRC patients involved in the circRNAs microarray experiment.

| Patient | Sex | Age | T | N | M | size(cm) |
| --- | --- | --- | --- | --- | --- | --- |
| #1 | Male | 66 | 3 | 0 | 0 | 6*4 |
| #2 | Male | 69 | 3 | 1 | 0 | 5*4 |
| #3 | Female | 72 | 3 | 2 | 1a | 8*7 |
