# Supplementary figures and images for: Circular RNA GLIS2 promotes colorectal cancer cell motility via activation of the NF-κB pathway
Source: Cell Death Dis. 2020 Sep 23;11(9):788. doi: 10.1038/s41419-020-02989-7 (PMC7511409; doi:10.1038/s41419-020-02989-7)

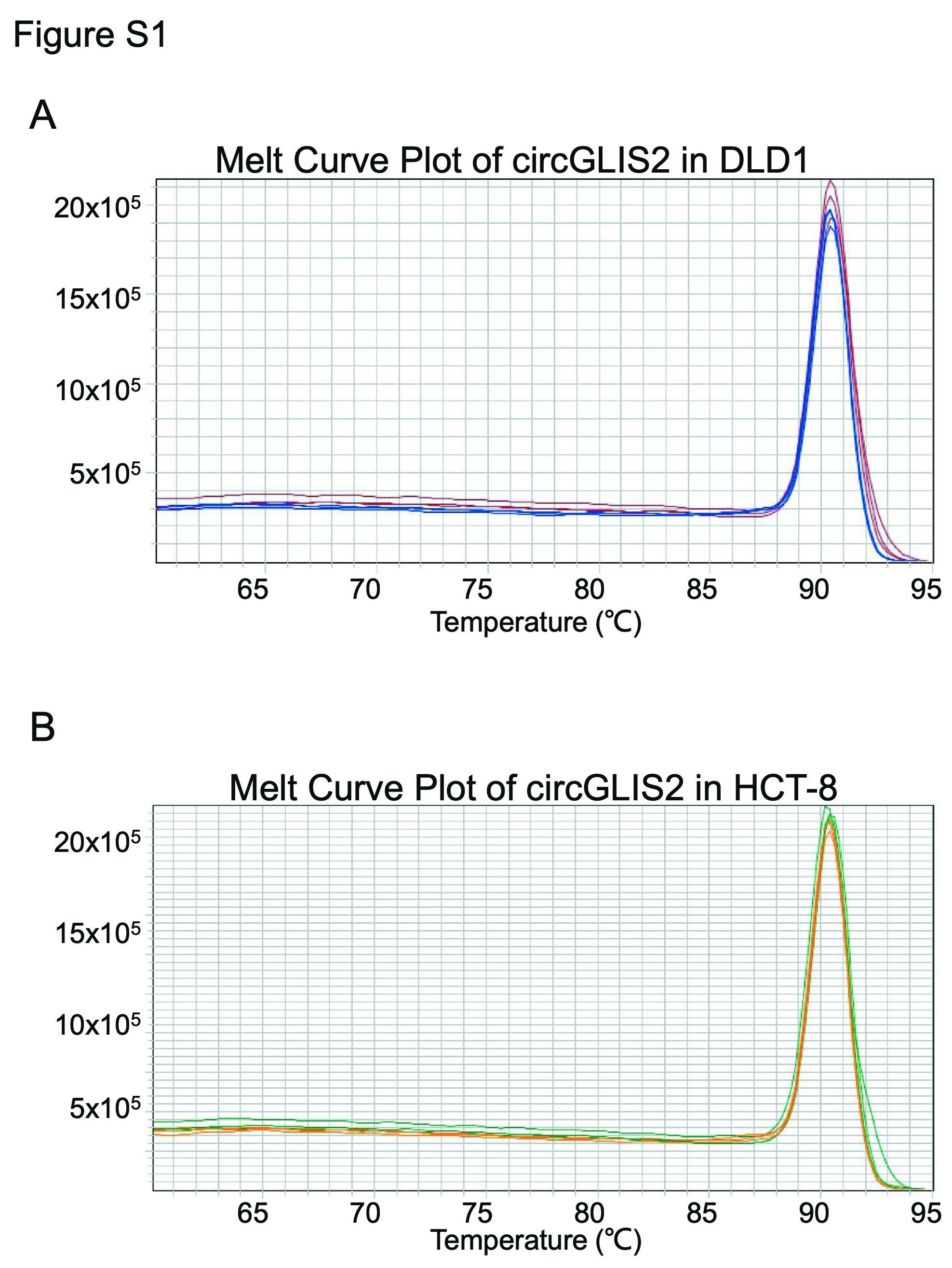

Supplement: Supplementary file 3 — figure S1 [file 41419_2020_2989_MOESM3_ESM.jpg]

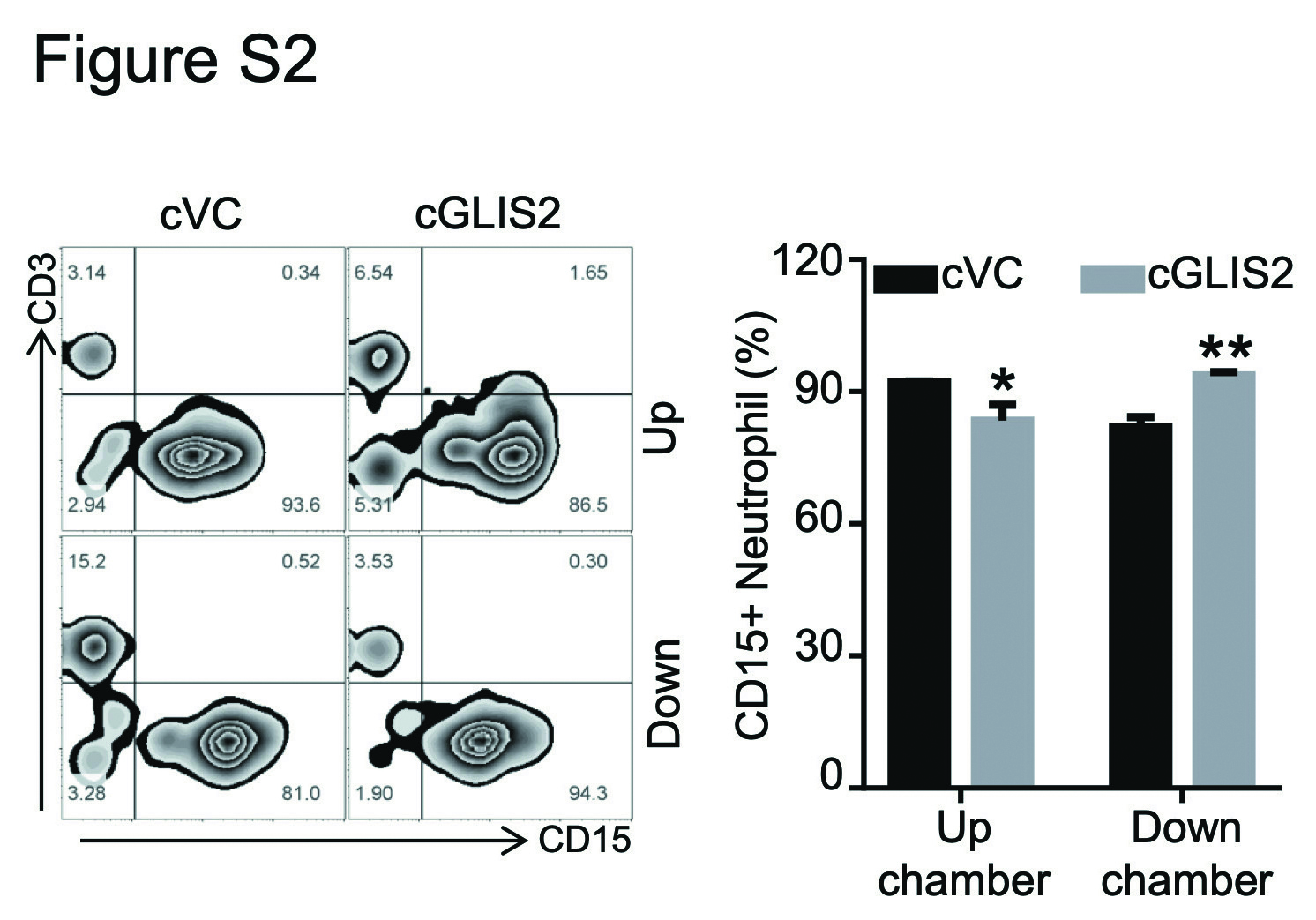

Supplement: Supplementary file 4 — figure S2 [file 41419_2020_2989_MOESM4_ESM.jpg]

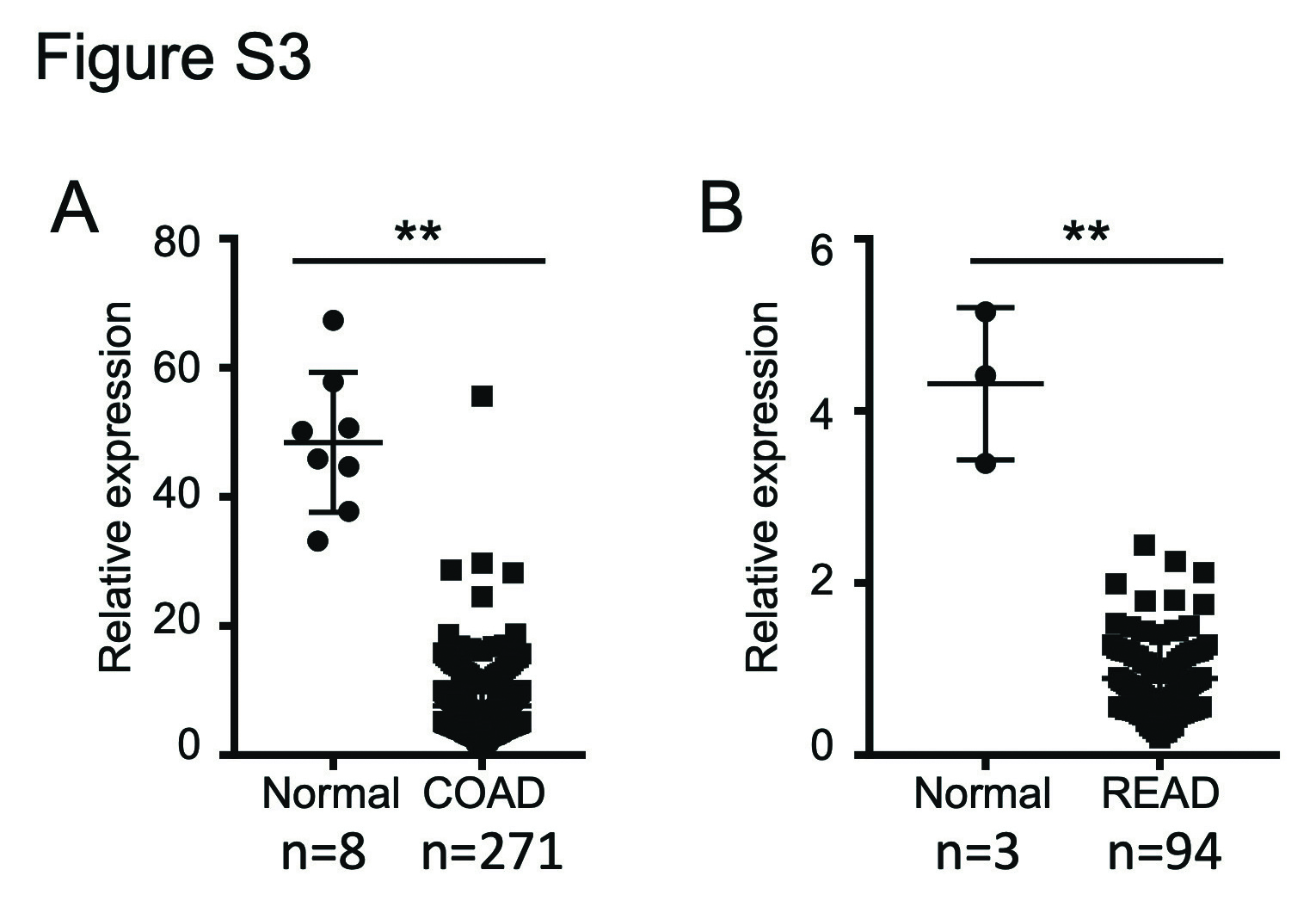

Supplement: Supplementary file 5 — figure S3 [file 41419_2020_2989_MOESM5_ESM.jpg]

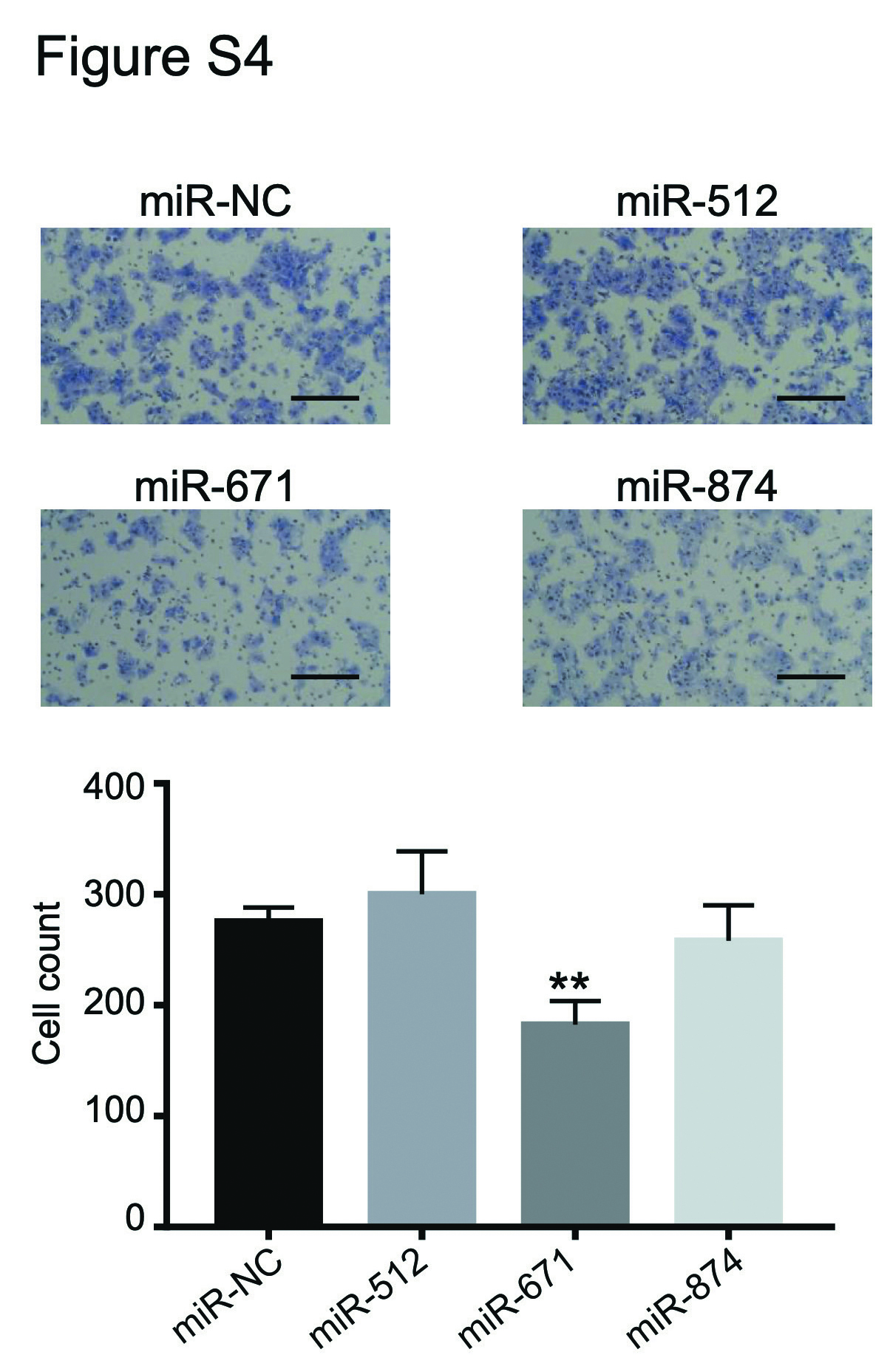

Supplement: Supplementary file 6 — figure S4 [file 41419_2020_2989_MOESM6_ESM.jpg]
